# Clinical characteristics and antibody responses to Omicron variants among pregnant women in China during the December 2022–April 2023 COVID-19 pandemic wave

**DOI:** 10.3389/fimmu.2026.1845051

**Published:** 2026-05-28

**Authors:** Ruijing Bao, Lanxin Ma, Rongrong Dai, Xinyu Liu, Nani Xu, He Fang, Jianmin Jiang, Haichang Yin, Hangjie Zhang

**Affiliations:** 1 School of Life Sciences and Agriculture and Forestry, Qiqihar University, Qiqihar, China; 2 Department of Prevention and Control of Infectious Disease, Zhejiang Provincial Center for Disease Control and Prevention, Hangzhou, China; 3 Department of Public Health and Preventive Medicine, Wuxi School of Medicine, Jiangnan University, Wuxi, China; 4 School of Public Health, Hangzhou Medical College, Hangzhou, China; 5 Xihu District Center for Disease Control and Prevention, Hangzhou, China; 6 Department of Clinical Laboratory, Zhejiang Provincial People’s Hospital, Hangzhou, Zhejiang, China; 7 Zhejiang Key Lab of Vaccines, Department of Prevention and Control of Infectious Disease, Zhejiang Provincial Center for Disease Control and Prevention, Hangzhou, China

**Keywords:** antibody response, clinical characteristics, COVID-19, inactivated vaccine, omicron, pregnancy

## Abstract

**Background:**

Clinical characteristics and humoral immune responses in pregnant women following natural SARS-CoV-2 Omicron infection remain unclear, particularly in those previously receiving inactivated vaccines, which may induce immune imprinting and affect cross-neutralization against emerging Omicron subvariants.

**Methods:**

This cross-sectional investigation was conducted in Zhejiang Province between December 2022 and April 2023. A total of 223 pregnant women with a gestational age of at least six weeks and 31 healthy non-pregnant women were recruited as research subjects and controls. Serum specimens were collected to determine anti-RBD IgG levels by ELISA, alongside pseudovirus neutralizing antibody titers against the prototype strain and Omicron BA.4/5, XBB.1.5 subvariants. Relevant clinical information was gathered through questionnaires, and multivariate regression analysis was applied to identify influencing factors of immune response characteristics.

**Results:**

Acute COVID-19 symptom profiles were comparable between pregnant women and non-pregnant healthy women (P > 0.05). Among questionnaire respondents, pregnant individuals had markedly prolonged symptom recovery, with a notably higher proportion taking over one week to recover (73.0% vs. 23.1%, P = 0.045), and presented a significantly higher medical consultation rate (73.0% vs. 15.4%, P < 0.001). For immune response, neutralizing antibody levels against the Omicron BA.4/5 showed no intergroup difference overall. By contrast, pregnant women exhibited significantly lower XBB.1.5 neutralizing antibody titers than controls, with geometric mean titers of 16.35 and 45.96 respectively (P < 0.05). Multivariate regression analysis indicated that SARS-CoV-2 infection during the Omicron epidemic was the major independent factor linked to elevated neutralizing antibody levels, while a BMI below 25.0 kg/m² was independently correlated with higher XBB.1.5 neutralizing antibody titers. Additionally, no significant differences in clinical manifestations or immune responses were found among women in different trimesters of pregnancy.

**Conclusion:**

In vaccine-primed pregnant women with natural Omicron infection, acute clinical manifestations and homologous humoral immunity against BA.4/5 were similar to non-pregnant controls, but symptom recovery was prolonged and cross-neutralization against XBB.1.5 was reduced. No trimester-specific differences were observed.

## Introduction

The global outbreak of coronavirus disease 2019 (COVID-19), caused by severe acute respiratory syndrome coronavirus 2 (SARS-CoV-2), has resulted in more than 760 million confirmed cases and nearly 7 million deaths, marking it as one of the most significant public health crises of the 21st century (1). Since its emergence in November 2021, the Omicron variant has exhibited increased transmissibility and enhanced immune evasion due to numerous mutations in its spike protein (2). These characteristics have enabled Omicron to rapidly overtake previous variants, such as Delta, establishing itself as the dominant lineage worldwide (3). While infection with Omicron is generally associated with reduced disease severity, the clinical implications and immune response profiles in vulnerable populations—particularly pregnant women—require further investigation (4, 5).

The continuous emergence of Omicron sublineages, such as BA.4/5 and XBB.1.5, and their enhanced immune evasion capabilities have significantly reduced the protective effects of neutralizing antibodies elicited by prior infection or vaccination (6, 7). Highly mutated lineages such as XBB exhibit substantial immune evasion, posing ongoing challenges even in populations with high vaccination coverage (8). These subvariants can largely evade neutralizing antibodies elicited by inactivated vaccination (9), which is the most widely used vaccine platform in China, posing a significant challenge to the immune protection of vulnerable populations, including pregnant women (10). This epidemiological context provided a critical opportunity to evaluate the clinical progression and immune response dynamics in pregnant women following natural Omicron infection.

While Omicron is generally considered less virulent than previous variants, recent cohort studies highlight that pregnant women remain uniquely vulnerable during the Omicron era, experiencing higher risks of adverse maternal and neonatal outcomes if inadequately protected by vaccination (5, 11). Notably, the widespread administration of inactivated vaccines in China has created a distinct immunological background compared to Western populations, which are primarily immunized with mRNA vaccines, potentially influencing post-infection immune response patterns (12). Recent serological evaluations in Chinese adults have underscored that hybrid immunity—derived from inactivated vaccine priming followed by Omicron breakthrough infection—induces unique neutralizing profiles and antibody-dependent cellular cytotoxicity (ADCC) responses (13, 14). However, most existing studies have focused on mRNA-vaccinated populations in Western countries, and systematic studies on clinical outcomes and cross-neutralizing antibody responses against Omicron subvariants in pregnant women primed with Chinese inactivated vaccines are still scarce.

In this study, we compared post-infection clinical symptoms and recovery outcomes between pregnant and non-pregnant women, determined pseudovirus neutralizing antibody geometric mean titers against the prototype strain as well as Omicron BA.4/5 and XBB.1.5 variants in pregnant women, analyzed differences in such antibody levels across different gestational trimesters, and further explored the effects of infection status, BMI, age, vaccination history and gestational stage on anti-RBD IgG concentrations and neutralizing antibody responses.

## Materials and methods

### Study design and participants

**Figure 1:**
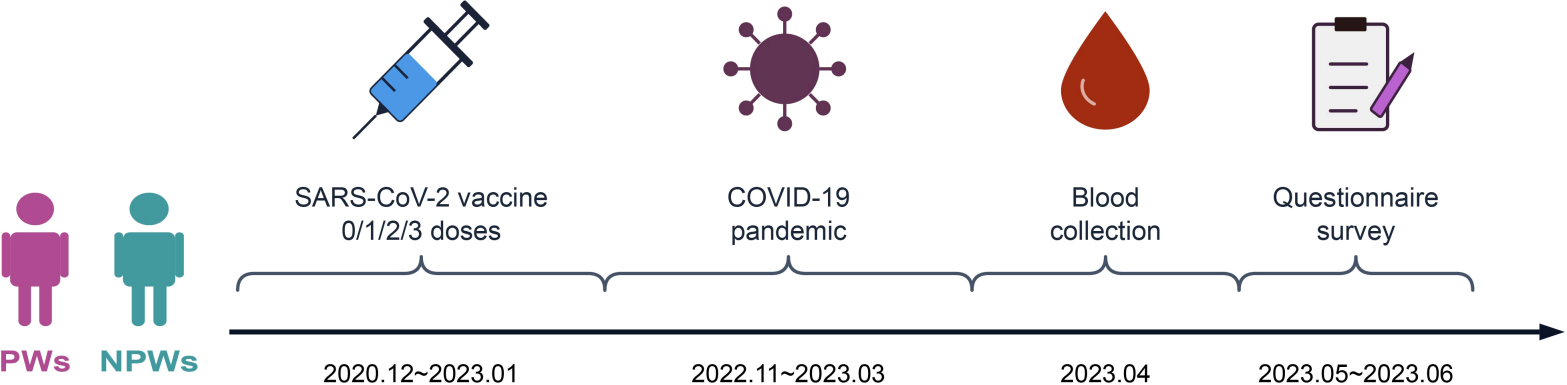
Study design. Flowchart outlining the overall study procedure for pregnant and non-pregnant participants. Depicted are the timeline of SARS-CoV-2 vaccination, the period of the COVID-19 Omicron epidemic wave, the time points for peripheral blood sample collection, and the follow-up questionnaire investigation arranged in chronological order.

This cross-sectional study was performed in Zhejiang Province, China, between December 2022 and April 2023, with the overall research timeline shown in **Figure 1**. The participants comprised 223 pregnant women at a gestational age of at least six weeks recruited from Zhejiang Provincial People’s Hospital and 31 healthy non-pregnant women with matched age and vaccination backgrounds enrolled from local communities; none of the subjects had experienced SARS-CoV-2 infection prior to the Omicron epidemic wave spanning December 2022 to April 2023.

SARS-CoV-2 infection status was defined as follows: participants were classified as infected if they reported a positive SARS-CoV-2 antigen or PCR test, or presented typical COVID-19-like symptoms accompanied by serological evidence of prior infection with anti-RBD IgG (BA.4/5) OD_450_–OD_630_ ≥ 0.165, while those without any infection history and with anti-RBD IgG values below 0.165 were classified as uninfected. All participants provided blood specimens in April 2023, and a standardized questionnaire was administered from May to June 2023 to collect information on COVID-19-related clinical symptoms and post-infection recovery conditions, as well as demographic indicators including age, body mass index, vaccination history, and gestational trimester among pregnant participants.

All participants provided written informed consent prior to enrollment, and the study protocol was approved by the Ethics Committee of Zhejiang Provincial People’s Hospital (approval number: KT2023025).

### Immunological and antibody assessments

For the neutralization assay, serum samples were initially diluted 1:8, followed by two-fold serial dilutions in 96-well plates. SARS-CoV-2 pseudovirus was incubated with diluted serum for 1 hour at 37 °C, and the mixture was then applied to Vero cell monolayers. After 20 hours of incubation, the transduction units were quantified using an EVOS M7000 automated live-cell fluorescence imaging system and analyzed with ImageJ software. The 50% pseudovirus neutralization titer (pVNT50) was calculated by nonlinear regression using GraphPad Prism. Results are reported as geometric mean titers of pVNT50 against each SARS-CoV-2 variant tested.

An enzyme-linked immunosorbent assay (ELISA) was used to assess the binding capacity of IgG antibodies in participant serum samples to the receptor-binding domain (RBD) of the SARS-CoV-2 Omicron BA.4/5 variant. Recombinant RBD protein matching the target antigen sequence was pre-coated onto microplate wells. Blank control wells (diluent only, without samples or detection antibody), positive controls, negative controls, and diluted test samples (100 μL each) were applied to designated wells. The sealed plate was incubated at 37 °C for 1 hour to facilitate the binding of IgG. Wells were then washed five times with at least 300 μL wash buffer (PBS containing 0.05% Tween-20) per cycle, with 30-second soaking intervals, to remove unbound components. After washing, 100 μL of enzyme-conjugated detection antibody was added to each well, followed by a 30-minute incubation at 37 °C. A subsequent wash step was performed, after which a tetramethylbenzidine (TMB) substrate solution was added, and the plate was incubated at 37 °C in the dark for 15 minutes. The reaction was terminated by adding 50 μL of the stop solution, and the absorbance was immediately measured at 450 nm and 630 nm using a microplate reader. Anti-RBD IgG levels were expressed as the difference in optical density (OD_450_–OD_630_).

### Statistical analysis

The categorical variables are presented as numbers and percentages, anti-RBD IgG levels with non-normal distribution were expressed as the median with interquartile range, and serum neutralizing antibody titers pVNT50 were expressed as GMT with 95% confidence interval and log10-transformed prior to analysis. We used unpaired t-test, paired t-test, and one-way analysis of variance with Tukey’s multiple comparisons test to analyze the log10-transformed antibody titers. The Mann-Whitney U test and Kruskal-Wallis test were used for non-normally distributed data. The Pearson chi-square test and Fisher’s exact test were performed to analyze categorical data. Multiple linear regression models were used for multivariable analyses, with variance inflation factor below 5 to exclude multicollinearity. All statistical tests were two-sided, and P-values < 0.05 were considered statistically significant. All analyses were conducted using R version 4.3.1 and GraphPad Prism version 9.

## Results

### Demographic characteristics

Baseline demographic and clinical features of all participants are summarized in **Table 1**. Of all pregnant women, 78.5% (175/223) were aged under 35 years and 21.5% (48/223) were 35 years or older, with an interquartile age range of 29–34 years. Among non-pregnant women, 54.8% (17/31) were younger than 35 years and 45.2% (14/31) were aged 35 years or above, with an interquartile range of 29–45 years. In terms of body mass index (BMI), 33.2% of pregnant women had a BMI below 25.0 kg/m² and 10.3% had a BMI of 25.0 kg/m² or higher; all non-pregnant women had complete BMI data, of whom 77.4% had a BMI under 25.0 kg/m² and 22.6% had a BMI ≥25.0 kg/m².

**Table 1:** Participant demographic characteristics.

Variable	Pregnant women (N = 223)	Non-pregnant women (N = 31)
Age, years		
<35	175(78.5)	17(54.8)
≥35	48(21.5)	14(45.2)
P25-P75	29-34	29-45
BMI (kg/m²)		
<25.0	74(33.2)	24(77.4)
≥25.0	23(10.3)	7(22.6)
Unknown	126(56.5)	—
Vaccine(s) administered		
Yes	212(95.1)	30(96.8)
No	11(4.9)	1(3.2)
Gestational stage		
First trimester	12(5.4)	—
Second trimester	65(29.1)	—
Third trimester	94(42.2)	—
unknown	52(23.3)	—

Data are presented as the number of participants (%). Statistical analyses were conducted using the Pearson Chi-squared test, or Fisher’s exact test where appropriate. Gestational stages are defined by gestational age as follows: first trimester (<14 weeks), second trimester (14–27 weeks), and third trimester (≥28 weeks).

Vaccination coverage was similar across the two groups, with 95.1% of pregnant women and 96.8% of non-pregnant women having received COVID-19 vaccines, and only a small fraction unvaccinated. All vaccinated pregnant women finished the full three-dose inactivated vaccine regimen. Gestational trimester data were only available for pregnant participants, with 5.4% in the first trimester, 29.1% in the second trimester, 42.2% in the third trimester, and 23.3% with unclear gestational stage.

### Analysis of COVID-19 clinical characteristics and associated factors

As presented in **Table 2**, clinical recovery differed notably between the two groups. Pregnant women had considerably longer symptom persistence, with 73.0% taking one week or more to recover, compared with merely 23.1% of non-pregnant women (P = 0.045). Pregnant women also had a much higher rate of medical attendance at 73.0%, versus 15.4% in the non-pregnant group (P<0.001). Furthermore, the time to negative conversion of SARS-CoV-2 antigen or PCR tests was markedly extended among pregnant women, 73.0% took at least one week to turn negative, while the figure was only 26.9% for non-pregnant women (P < 0.001). Of these followed-up participants, no COVID-19-related adverse neonatal outcomes occurred; 87.5% (56/64) had full-term vaginal delivery, while 12.5% (8/64) underwent full-term cesarean section for indications unrelated to COVID-19.

**Table 2:** Symptoms of COVID-19 in the questionnaire-responsive subgroup.

Variable	Pregnant women (N = 74)	Non-pregnant women (N = 26)	P
COVID-19 infection			0.024*
Yes	179(80.3)	30(96.8)	
No	44(19.7)	1(3.2)	
General symptom			0.053*
Yes	73(98.6)	23(88.5)	
No	1(1.4)	3(11.5)	
Respiratory symptom			0.111
Yes	55(74.3)	15(57.7)	
No	19(25.7)	11(42.3)	
Digestive tract symptom			0.260*
Yes	0(0)	1(3.8)	
No	74(100)	25(96.2)	
Fever			0.887
<38 °C	21(28.4)	7(26.9)	
≥38 °C	53(71.6)	19(73.1)	
Muscular soreness			0.097
Yes	53(71.6)	14(53.8)	
No	21(28.4)	12(46.2)	
Symptom recovery time			0.045
<1 week	20(27.0)	20(76.9)	
≥1 week	54(73.0)	6(23.1)	
Medical visits after COVID-19 infection			<0.001
Yes	54(73.0)	4(15.4)	
No	20(27.0)	22(84.6)	
Time of SARS-CoV-2 antigen or PCR turns to negative			<0.001
<1 week	20(27.0)	19(73.1)	
≥1 week	54(73.0)	7(26.9)	

Data are presented as number of participants (%). Symptom categorization was defined: general symptoms comprised fatigue, chills, and weakness; respiratory symptoms comprised cough, sore throat, nasal congestion, and rhinorrhea; gastrointestinal symptoms comprised nausea, vomiting, and diarrhea. Statistical analyses were performed using the chi-square test and Fisher’s exact test. *P-values were calculated using Fisher’s exact test

Univariate analysis revealed no significant associations between demographic factors and clinical manifestations in SARS-CoV-2-infected pregnant women. Stratification by age (<35 vs. ≥35 years), BMI (<25.0 vs. ≥25.0 kg/m²), and vaccination status demonstrated no statistically significant disparities in infection rates, general symptoms, respiratory symptoms, fever ≥38 °C, or muscular soreness (all P > 0.05). Furthermore, analysis by gestational stage indicated no significant associations with the incidence of general symptoms, respiratory symptoms, muscular soreness, or fever ≥38 °C across the three trimesters (all P > 0.05) (**Table 3**).

**Table 3:** Association between participant characteristics and COVID-19 symptoms in pregnant women.

Variable	COVID-19 infection		General symptom		Respiratory symptom		Fever (≥38 °C)		Muscular soreness	
	N (%)	P	N (%)	P	N (%)	P	N (%)	P	N (%)	P
Age,years										
<35	139(77.7)	0.547	54 (100.0)	0.270	41(75.9)	1.000*	38 (70.4)	0.695	41(75.9)	0.177
≥35	40(22.3)		19 (95.0)		14(70.0)		15 (75.0)		12(60.0)	
BMI (kg/m²)										
<25.0	68(78.2)	0.178	60 (98.4)	1.000*	48 (78.7)	0.083	42 (68.9)	0.326	41(67.2)	0.094
≥25.0	19(21.8)		13 (100.0)		7 (53.8)		11 (84.6)		12 (92.3)	
Vaccine(s) administered										
Yes	170(95.0)	1.000*	72 (98.6)	1.000*	54(74.0)	1.000*	52(71.2)	1.000*	52(71.2)	1.000*
No	9(5.0)		1(100.0)		1(100.0)		1(100.0)		1(100.0)	
Gestational stage										
First trimester	9(6.4)	0.460	4(100.0)	0.428	4(100.0)	0.405	3(75.0)	0.208	2(50.0)	0.692
Second trimester	51(36.4)		21(95.2)		15(71.4)		14(66.7)		14(66.7)	
Third trimester	80(57.1)		31(100.0)		25(80.6)		24(87.1)		22(71.0)	

Data are presented as the number of participants (%). Statistical analyses were conducted using the Pearson Chi-squared test. *P-values were calculated using Fisher’s exact test.

### Analysis of immunogenicity

**Figure 2:**
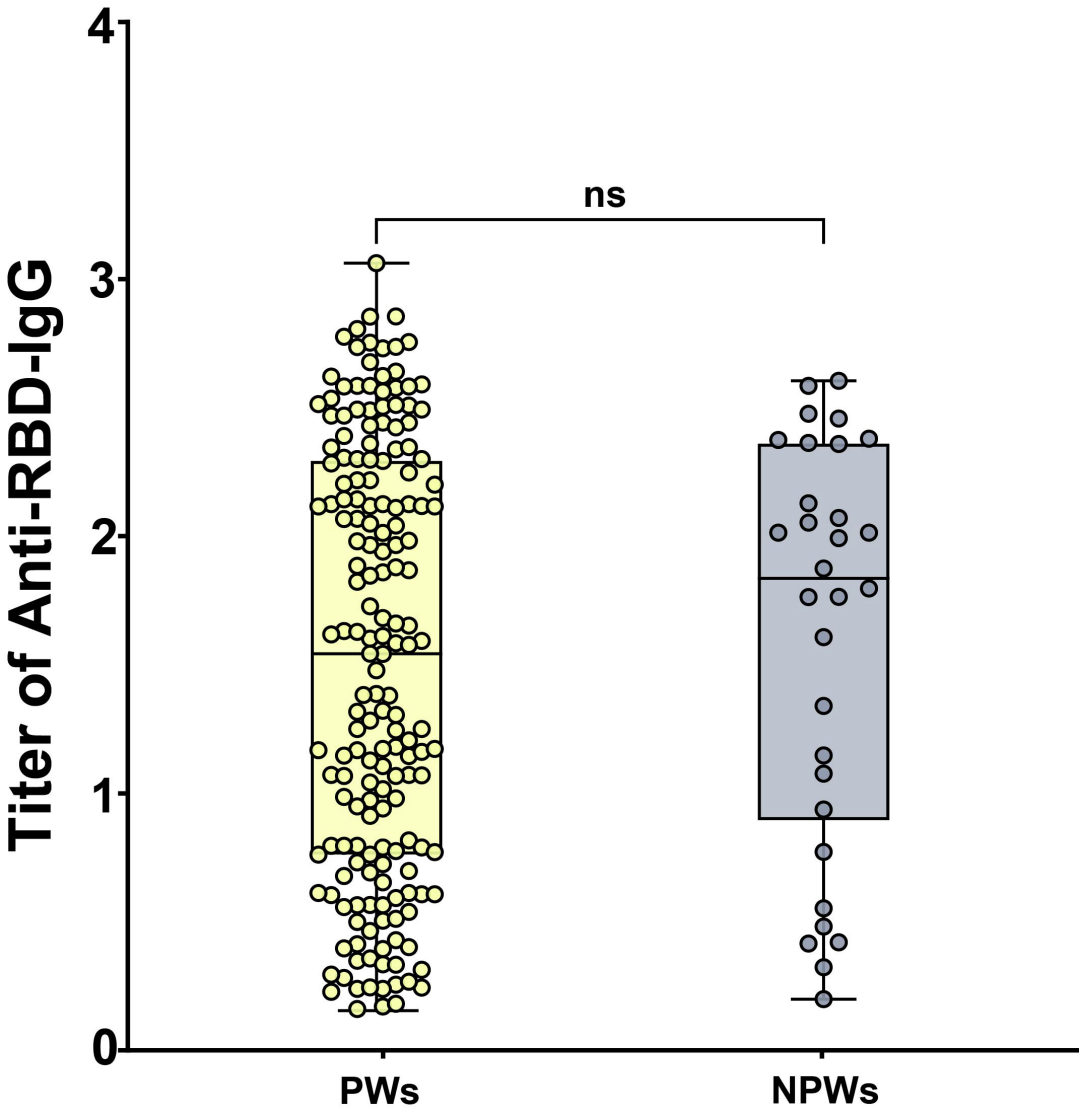
Anti-SARS-CoV-2 RBD IgG antibody levels in pregnant and non-pregnant women. Anti-SARS-CoV-2 RBD IgG (BA.4/5) antibody levels in pregnant and non-pregnant women. Antibody levels were quantified by ELISA and presented as optical density values (OD450–OD630). Each dot denotes an individual sample. The middle horizontal line shows the median value, and whiskers indicate the interquartile range. Between-group comparisons were performed using the two-tailed Mann-Whitney U test. ns, no significant difference, P > 0.05.

Anti-SARS-CoV-2 RBD IgG (BA.4/5) were comparable between the two study groups (**Figure 2**). Specifically, the median titer was 1.6 (IQR: 0.8–2.3) in pregnant women, which did not differ significantly from 1.9 (IQR: 1.0–2.4) in non-pregnant women (P = 0.615).

**Figure 3:**
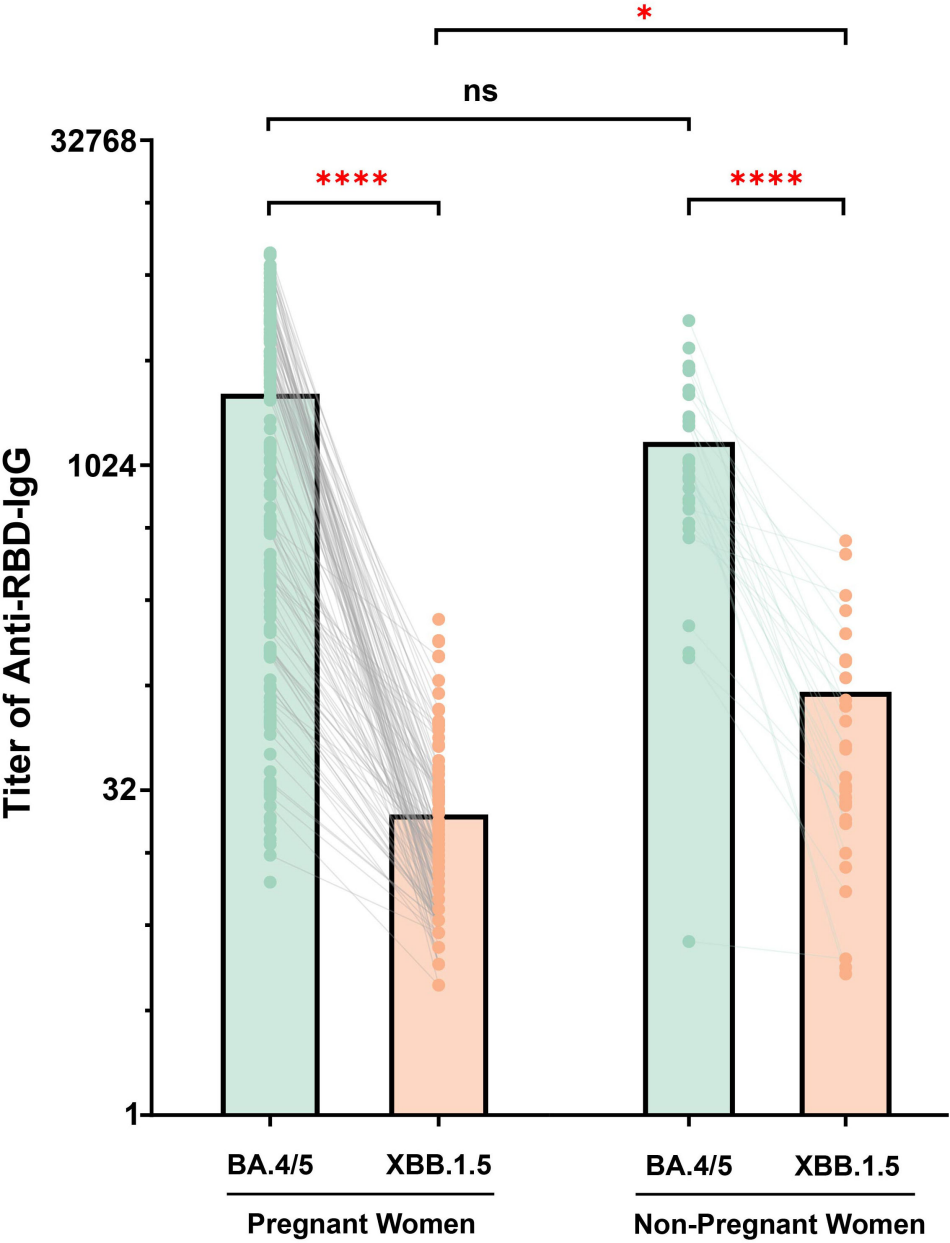
Pseudovirus neutralizing antibody titers against Omicron BA.4/5 and XBB.1.5 in pregnant and non-pregnant women. Neutralizing antibody titers (pVNT50) against Omicron BA.4/5 and XBB.1.5 subvariants in pregnant women and non-pregnant women. The y-axis is shown on a log10 scale. Each symbol corresponds to an individual participant. Bars denote geometric mean titers (GMT) with 95% confidence intervals (CI). Intergroup differences were assessed by unpaired t-test using log10-transformed pVNT50 values; intragroup comparisons between the two Omicron subvariants were performed via paired t-test on log10-transformed data. *P < 0.05; ****P < 0.0001; ns, no significant difference (P > 0.05).

Pseudovirus-based neutralization assays for Omicron BA.4/5 and XBB.1.5 were conducted in both pregnant and non-pregnant participants (**Figure 3**). Among pregnant women, the pVNT50 GMTs were 704.8 (95% CI: 104.0–3899.0) for BA.4/5 and 16.35 (95% CI: 8.00–28.75) for XBB.1.5, with a marked difference detected between the two variants (P < 0.001). In non-pregnant women, the corresponding pVNT50 GMTs were 812.1 (95% CI: 575.3–1699.0) and 45.96 (95% CI: 22.65–121.0), also showing a significant inter-variant difference (P < 0.001). Additionally, neutralizing activity against XBB.1.5 differed substantially between the two groups, with pregnant women presenting notably lower antibody levels than non-pregnant controls (P < 0.05).

**Figure 4:**
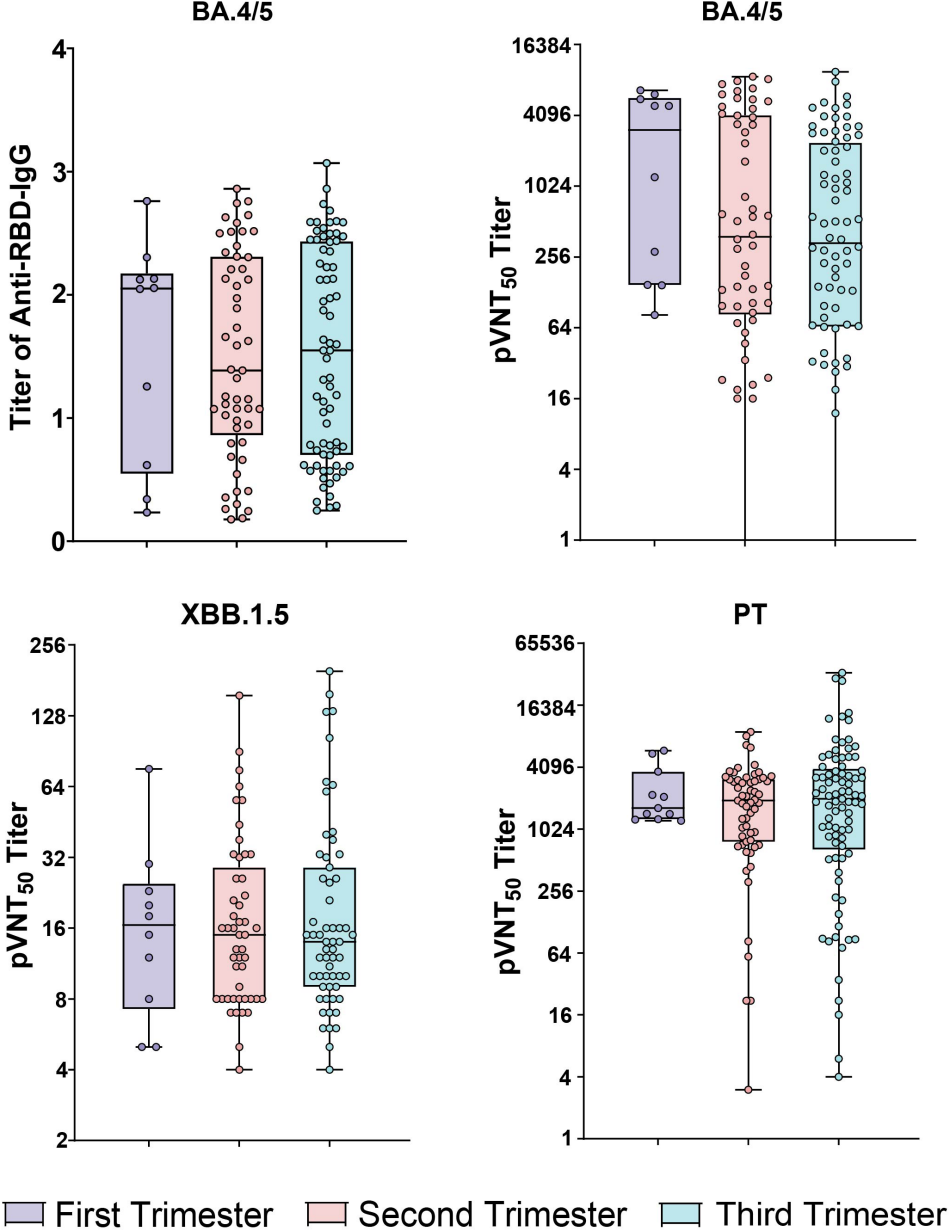
Antibody responses to SARS-CoV-2 variants in pregnant women across gestational trimesters. Levels of anti-SARS-CoV-2 BA.4/5 RBD IgG (OD450–OD630 absorbance) and pVNT50 neutralizing titers against Omicron BA.4/5, XBB.1.5, and the prototype SARS-CoV-2 strain were analyzed in pregnant women grouped by first (n=12), second (n=65), and third (n=94) trimesters. Box plots display the interquartile range, with the median denoted by the central horizontal line. Inter-trimester differences were evaluated by one-way ANOVA on log10-transformed data, followed by Tukey’s multiple comparisons test.

To further explore whether advancing pregnancy affects immune responses, pregnant participants were stratified by gestational trimester (**Figure 4**). Anti-RBD IgG (BA.4/5) concentrations showed no obvious variation across the three trimesters (P = 0.840). Likewise, pseudovirus neutralizing titers against BA.4/5, XBB.1.5 and the prototype strain remained comparable among different gestational stages, with no statistically significant differences noted (P = 0.645, 0.291, and 0.160, respectively).

### Analysis of influencing factors

Regarding recent SARS-CoV-2 infection, individuals infected during the Omicron wave had markedly higher neutralizing antibody titers against BA.4/5, with a GMT of 968.6 (95% CI: 727.2–1290.0), versus 55.7 (95% CI: 26.6–116.3) in uninfected participants (P < 0.001). Likewise, XBB.1.5 neutralizing antibody levels were substantially higher in infected individuals (GMT: 17.22, 95% CI: 15.0–19.8) than in uninfected counterparts (GMT: 8.4, 95% CI: 6.5–10.9) (P = 0.005).

Univariate analysis revealed that age, vaccination status and gestational trimester exerted no significant influence on anti-RBD IgG, BA.4/5 or XBB.1.5 antibody concentrations (all P > 0.05). By contrast, BMI was significantly correlated with neutralizing antibody responses to both BA.4/5 (P = 0.033) and XBB.1.5 (P = 0.046) (**Table 4**).

**Table 4:** Association between participant characteristics and antibody levels in pregnant women.

Variable	Anti-RBD IgG		BA.4/5		XBB.1.5		PT	
	Titer	P	GMT	P	GMT	P	GMT	P
Age,years								
<35	1.02 (0.3, 2.2)	0.080	729.5 (525.2, 1013.0)	0.662	16.7 (14.3,19.4)	0.549	1240.0 (964.1,1594.0)	0.700
≥35	1.49 (1.05, 2.4)		626.1 (337.6,1161.0)		15.0 (11.3,20.0)		1377.0 (865.4,2190.0)	
BMI (kg/m²)								
<25.0	1.19 (0.6, 2.2)	0.290	878.0 (559.4,1378.0)	0.033	19.3 (15.7,23.9)	0.046	1698.0 (1244.0,2318.0)	0.816
≥25.0	1.1 (0.5, 1.5)		2313.0 (1292.0,4141.0)		12.9 (9.3,18.0)		1571.0 (817.6, 3019)	
Vaccine(s) administered								
Yes	1.1 (0.3, 2.1)	0.324	691.0 (512.4,932.0)	0.605	16.4 (14.3,18.9)	0.757	1289.0 (1032.0, 1610.0)	0.530
No	1.7 (0.8, 2.3)		942.0 (288.6,3075.0)		15.0 (7.9, 28.6)		943.3 (242.3, 3672.0)	
COVID-19 infection								
Yes	1.6 (0.8, 2.3)	<0.001	968.6 (727.2,1290.0)	<0.001	17.22 (15.0,19.8)	0.005	1751.0 (1472.0, 2084.0)	<0.001
No	0.06 (0.03, 0.09)		55.7(26.6,116.3)		8.4 (6.5,10.9)		289.7 (130.8, 642.0)	
Gestational Stage								
First trimester	1.9 (0.2, 2.4)	0.840	1517.0 (676.6,3401.0)	0.057	15.0 (9.8,22.9)	0.828	2002.0 (1530.0,2619.0)	0.544
Second trimester	1.3 (0.3, 2.2)		476.9 (270.4,840.9)		16.5 (13.1, 20.7)		1313.0 (897.8, 1920.0)	
Third trimester	1.1 (0.4, 2.0)		483.5 (313.3,746.3)		17.2 (13.6,21.8)		1318.0 (890.6, 1949.0)	

Data for anti-SARS-CoV-2 RBD IgG levels are presented as the median and interquartile range (IQR), and comparisons were performed using the Mann-Whitney U test (for two groups) or Kruskal-Wallis test (for three groups). Data for pseudovirus neutralization titers (pVNT50) against Omicron subvariants (BA.4/5, XBB.1.5) and the prototype strain (PT) are presented as geometric mean titers (GMT) with 95% confidence intervals (CI). Statistical significance for pVNT50 was determined using unpaired t-tests or one-way ANOVA following log10 transformation. A P value < 0.05 was considered statistically significant.

Due to limited serum availability or assay invalidation for certain samples, the effective sample sizes (n) vary across specific neutralization assays.

Multivariable regression analysis was further applied to identify independent determinants of immune response. Recent Omicron-era SARS-CoV-2 infection was independently linked to elevated RBD-specific IgG levels (B = 1.5; 95% CI: 1.21–1.69), and also served as a positive predictor of higher neutralizing titers against the prototype strain (B = 2.6; 95% CI: 1.82–3.36) and BA.4/5 variant (B = 3.1; 95% CI: 2.05–4.07). Additionally, participants with a BMI below 25 kg/m² presented significantly higher XBB.1.5 neutralizing antibody levels than those with a BMI≥25 kg/m² (B = 0.6; 95% CI: 0.01-1.11).

## Discussion

As SARS-CoV-2 continues to circulate and undergoes continuous antigenic evolution, defining the clinical course and immune protection landscape in vulnerable populations such as pregnant women, remains a core objective of global public health. In the present cross-sectional study, we systematically characterized the clinical manifestations and humoral immune responses following Omicron infection among pregnant women primed with inactivated COVID-19 vaccines, using non-pregnant women as controls. Our results indicate that although pregnant women displayed distinct patterns in post-infection symptom resolution and cross-neutralizing antibody breadth, their overall acute clinical presentation and homologous humoral immunity against the circulating Omicron BA.4/5 subvariant were largely comparable to those of non-pregnant women. These observations imply that pregnancy-related physiological and immunological adaptations do not severely compromise the maintenance of humoral immune memory against SARS-CoV-2 (15).

In terms of acute clinical manifestations, the frequencies of fever, respiratory symptoms, and myalgia were similar between pregnant and non-pregnant groups, suggesting a comparable initial disease phenotype upon Omicron breakthrough infection. Despite similar acute symptom profiles, our data revealed notable disparities in disease recovery trajectories. Pregnant women exhibited a significantly higher proportion of prolonged symptom resolution and a markedly elevated rate of medical consultation relative to non-pregnant controls. These findings underscore the need for enhanced clinical surveillance and supportive care for pregnant individuals in the post-acute phase of COVID-19, even when initial symptoms are mild (16).

From an immunological perspective, levels of anti-RBD IgG and neutralizing antibodies against the dominant Omicron BA.4/5 subvariant were statistically indistinguishable between pregnant and non-pregnant participants. Multivariate regression analysis further verified that recent natural infection during the Omicron epidemic wave was the strongest independent factor associated with elevated neutralizing antibody titers against BA.4/5. This finding reinforces the critical role of hybrid immunity (inactivated vaccination plus breakthrough infection) in eliciting robust homologous neutralization against currently circulating strains in the Chinese population (17).

Accumulating evidence highlights the rapid antigenic drift of SARS-CoV-2; emerging subvariants including Omicron XBB and BA.2.86/JN.1 lineages exhibit substantially enhanced immune evasion compared with BA (18). Pregnant women displayed significantly lower neutralizing geometric mean titers against XBB.1.5 compared with non-pregnant controls, indicating a more restricted ability to cross-neutralize antigenically divergent Omicron sublineages. As highlighted by Yang et al., such evolutionary trends may further narrow the already limited cross-neutralization breadth in populations primed with inactivated vaccines, placing pregnant women at potentially elevated risk of breakthrough infection by newly emerging variants.

We also evaluated whether gestational stage modulates humoral immune responses. Previous studies have yielded inconsistent results: Atyeo et al. reported stable anti-Spike antibody titers across trimesters in pregnant women receiving mRNA COVID-19 vaccines (19), while Yang et al. suggested that gestational age at vaccination may affect maternal antibody levels (20). Our data align with the former, demonstrating no significant differences in anti-RBD IgG levels or neutralizing activity against the prototype strain, BA.4/5, or XBB.1.5 among first, second, and third trimesters. These results suggest that trimester-specific immunological shifts do not meaningfully alter the magnitude of humoral immunity against SARS-CoV-2 in inactivated vaccine-primed pregnant women (21).

Our study has several limitations. First, the relatively low questionnaire response rate among pregnant women, small sample size of the non-pregnant control group, and high missing data for BMI and gestational stage may reduce the statistical power and generalizability of subgroup analyses. Second, due to the rapid emergence of novel Omicron subvariants, our neutralization panel did not include recently circulating strains such as JN.1, XDV, and NB.1.8.1. Nevertheless, individuals infected during the Omicron BA.4/5 epidemic wave that spanned December 2022 to April 2023 exhibited a predictable decline in neutralizing antibody titers against these newly emerged subvariants (22). Third, our evaluation was restricted to humoral responses, we did not assess cellular immune parameters including SARS-CoV-2-specific T-cell and memory B-cell responses, which contribute substantially to antiviral defense and long-term immune memory.

In conclusion, our findings demonstrate that pregnant women infected with Omicron exhibit similar acute clinical symptoms and homologous humoral immunity against the BA.4/5 subvariant to non-pregnant controls, but experience prolonged symptom recovery and reduced cross-neutralizing capacity against the highly divergent XBB.1.5 subvariant. No significant differences in clinical manifestations or humoral immune responses were observed across different gestational trimesters, indicating that trimester-specific physiological changes do not substantially impact the immune response to Omicron infection in this vaccinated population. These results highlight the need for continued clinical monitoring of pregnant women during post-infection recovery and underscore the importance of further research to address the reduced cross-protection against emerging Omicron subvariants in this vulnerable group.

## Data Availability

The raw data supporting the conclusions of this article will be made available by the authors, without undue reservation.
